# Deep Learning with Discriminative Margin Loss for Cross-Domain Consumer-to-Shop Clothes Retrieval

**DOI:** 10.3390/s22072660

**Published:** 2022-03-30

**Authors:** Pendar Alirezazadeh, Fadi Dornaika, Abdelmalik Moujahid

**Affiliations:** 1 Department of Informatics, University of the Basque Country, 20008 Donostia-San Sebastian, Spain; pendar.alirezazadeh@ehu.eus; 2 School of Computer and Information Engineering, Henan University, Kaifeng 475000, China; 3 Ikerbasque, Basque Foundation for Science, Plaza Euskadi, 5, 48009 Bilbao, Spain; 4 Department of Mathematics, University of the Basque Country, 48080 Bilbao, Spain; abdelmalik.moujahid@ehu.eus

**Keywords:** cross-domain fashion retrieval, margin-based loss function, adaptive margin, deep learning, discriminative analysis

## Abstract

Consumer-to-shop clothes retrieval refers to the problem of matching photos taken by customers with their counterparts in the shop. Due to some problems, such as a large number of clothing categories, different appearances of clothing items due to different camera angles and shooting conditions, different background environments, and different body postures, the retrieval accuracy of traditional consumer-to-shop models is always low. With advances in convolutional neural networks (CNNs), the accuracy of garment retrieval has been significantly improved. Most approaches addressing this problem use single CNNs in conjunction with a softmax loss function to extract discriminative features. In the fashion domain, negative pairs can have small or large visual differences that make it difficult to minimize intraclass variance and maximize interclass variance with softmax. Margin-based softmax losses such as Additive Margin-Softmax (aka CosFace) improve the discriminative power of the original softmax loss, but since they consider the same margin for the positive and negative pairs, they are not suitable for cross-domain fashion search. In this work, we introduce the cross-domain discriminative margin loss (DML) to deal with the large variability of negative pairs in fashion. DML learns two different margins for positive and negative pairs such that the negative margin is larger than the positive margin, which provides stronger intraclass reduction for negative pairs. The experiments conducted on publicly available fashion datasets DARN and two benchmarks of the DeepFashion dataset—(1) Consumer-to-Shop Clothes Retrieval and (2) InShop Clothes Retrieval—confirm that the proposed loss function not only outperforms the existing loss functions but also achieves the best performance.

## 1. Introduction

Finding fashion images is one of the most sought-after applications in E-commerce. This application allows customers to discover their favorite clothes in online stores, and it can be considered as an important step for future applications in the fashion industry, such as outfit recommendations, i.e., customers search for an outfit after retrieving their desired clothes.

Finding items in online stores using customer photos based solely on their visual appearance has proven to be a major challenge for the computer vision community. Since customer and store images come from different heterogeneous domains, this problem is referred to as a cross-domain problem in apparel search. The quality of shooting equipment, lighting conditions, human body posture, and viewing angle are the main factors that explain the large visual differences between photos of customers and fashion images taken by professional photographers. The same clothes can look different under different circumstances such as light, different situations, or poses. In contrast, different clothes can appear visually similar.

Over the past decade, there has been considerable progress in garment search between consumers and stores using convolutional neural networks (CNNs) [1,2,3,4,5,6,7,8,9,10,11,12,13,14,15,16,17]. Using complex neural networks with a high number of layers, previous methods attempted to extract powerful features and improve retrieval performance. However, as the number of layers in the neural network increases, the intuitive low-level texture information of the clothing images is lost, while the abstract high-level semantic information is preserved, which is only suitable for image classification tasks, but not for cross-domain clothing searches [15]. Moreover, most of the existing methods use Triplet Loss to converge neural networks. Triplet Loss is specifically defined for the face-recognition problem. Human face images are always well-structured, have fixed image sizes, and differ only slightly from each other. Compared to face images, the cross-domain clothing images always have a large variety of different categories and clothing styles (significant intraclass differences), so the Triplet Loss is not suitable for cross-domain clothing retrieval [15].

**Figure 1:**
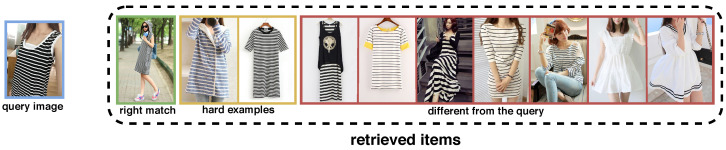
Example of consumer-to-shop clothes retrieval, which includes a query image (with a blue frame) and the 10 closest gallery images. The green frame represents the correct match, while the yellow examples represent hard examples, and the red frames represent items that differ from the query. As can be seen, the hard examples have many similarities with the query image. The slight superficial difference causes the images to be retrieved in the wrong way, which leads to system performance degradation.

Another challenge that has not been explicitly addressed is the small visual differences between certain garments (e.g., jeans and pants) that lead to unexpected garments being found and and customers being dissatisfied. Small visual differences lead to hard examples being found that have small visual differences from the query image, but do not match (see Figure 1).

In this work, we approach this problem by introducing a novel loss function that enforces a small intraclass distance and increases the distance between input pairs that are classified as dissimilar. Margin-based loss functions are typically motivated as approximations to upper bounds on misclassification loss. Contrastive loss and Triplet Loss are used by Siamese networks to extract discriminative features. These losses are based on metric distances and require a large number of utility pairs or triplet samples to obtain an optimal solution. Therefore, they are time-consuming and have poor performance on data from different domains with unbalanced features. Recently, much attention has been paid to softmax-based loss functions. Some researchers have optimized softmax and introduced margin-based softmax loss functions for discriminative analysis. Margin-based softmax losses such as Additive Margin-Softmax (aka CosFace) [18] normalized the feature and weight vectors by $l_{2}$-normalization to transform the angular margin of Softmax to the cosine margin, to improve the discriminative power of the original softmax loss. They varied the decision margin in the cosine space to modify intraclass and interclass variances, but since they consider the same margin for the positive and negative pairs, they are not suitable for cross-domain fashion search. We prefer a larger margin for negative pairs to strongly squeeze the intraclass variations of negative classes.

To achieve this goal, we propose a novel loss function for cross-domain search for clothes between consumers and stores, which we call Cross-Domain Discriminative Margin Loss (DML). DML learns two different cosine margins for positive and negative pairs to maximize the decision boundary and compact the negative decision margin in cosine space. Specifically, we make the margin *m* specific and learnable for each class and train the CNN directly. Formally, we define the positive margin $m_{p}$ and the negative margin $m_{n}$, such that the decision boundary is given by $\cos\left(\theta_{1}\right)-m_{p}=\cos\left(\theta_{2}\right)$ and $\cos\left(\theta_{1}\right)-m_{n}=\cos\left(\theta_{2}\right)$ for positive and negative classes, respectively, where $\theta_{i}$ is the angle between the feature and the weight of class *i*. In the experiments, we show that DML is superior to the Margin-based Softmax baseline methods. The Siamese networks are trained with DML to learn discriminative deep features for finding similar images. After training, the fashion-retrieval problem between consumers and stores is formulated as an asymmetric (single-to-multiple) matching problem. These features are input to the similarity distance metric to perform pairwise matching between customer and store images. Then, the top-ranked results are displayed to the customer.

The main contributions of the proposed work can be summarized as follows:

- A cross-domain discriminative loss function, called DML, is proposed to learn deep discriminative features for customer-to-shop fashion search.
- DML learns a larger margin for the negative class compared to the positive class to increase the variation between classes and reduce the negative class.
- The proposed approach achieves the best performance on consumer-to-shop fashion retrieval datasets, including DeepFashion [16] and DARN [17].

The rest of the paper is organized as follows. Section 2 introduces the related work. Section 3 describes our proposed method. Section 4 presents the experimental results obtained on two real fashion datasets. Finally, the discussion and conclusions are presented in Section 5 and Section 6, respectively.

## 2. Related Work

### 2.1. Fashion Retrieval

Over the past decade, consumer-to-shop image searches in stores have been widely studied [1,2,3,4,5,6,7,8,9,10,11,12,13,14,15,16,17]. Ref. [3] proposed the concept of cross-domain clothing search. Using human posture estimation, they estimated the human body area, extracted 30 regions of human body, and obtained the local features of clothing images, which can reduce the image differences due to cross-domain clothing images. They used a local feature-matching method and implemented cross-domain garment search through a two-stage sparse coding method. Although using the human posture estimation technique to extract local features is an intelligent solution to the cross-domain problem, this technique sometimes fails to detect regions of the human body based on different clothing postures. Therefore, the extracted irrelevant features may reduce the retrieval performance. Another study, ref. [5], proposed a novel region representation method to reduce the influence of complex and cluttered background environments. A binary spatial appearance mask was used to constrain the human body regions obtained by the pose-estimation algorithm. The methods based on the pose-estimation algorithm have the limitation that the same points must be visible in the whole image. Otherwise, the local features of different parts of the human body would be compared in cross-domain clothing images, which would lead to poor results. With the rapid development of convolutional neural networks (CNNs) in recent years, traditional methods of clothing analysis have been replaced by neural network models. In [1], the concept of precise cross-scene search to cope with this shift was proposed, with the goal of finding the exact same item on the shopping website when shopping online. They reduced the domain difference by removing the background of consumer images, which is one of the most critical sources of appearance variation, and using object proposals to select foreground items. Using pairwise mixed images from both domains, they trained deep similarity learning methods for the task of accurate street-to-store search. However, the object detectors do not work for complex gestures and the performance of deep similarity learning is sensitive to the introduction of pairwise images, which is a very time-consuming process according to the limited data. Dual attribute perceptual ranking network based on two fully independent branches (DARN) [17] has used feature learning for different scene domains integrating attribute and visual similarity constraints simultaneously. DARN uses two CNN-based branches for each of two domains and projects them into a common embedding space. Then, the output features of each subnetwork are concatenated and fed into the triplet ranking loss of the two subnetworks. Since the cross-domain clothing images have a large variety of different categories and clothing styles, the differences between the image pairs are very large and the Triplet Loss does not work well. FashionNet, proposed by [16], learns clothes retrieval by jointly predicting clothing attributes and landmark features, and applies the network to cross-scenario services for the DeepFashion dataset. FashionNet focuses on image keypoint localization by using the registered keypoints and image attribute information, which requires a lot of labor and also a lot of time to mark the keypoints of clothing images. Another study, [4], proposed a deep Siamese network with a modified contrastive loss and multitask fine-tuning method that trains a common model for all categories simultaneously. The Siamese network is directly trained for object detection/classification and then used for similarity estimation. On the other hand, contrastive loss attempts to make binary decisions about whether two images are similar, but cannot capture fine-grained similarity. Moreover, the common branch at the bottom of the network has learned features without considering higher-level semantic information. The authors of [6] used attribute labels to pay more attention to local discriminative regions. They employed attention mechanisms in global feature aggregation to focus network training on the clothes themselves, effectively neglecting the influence of background noise. However, their method relies heavily on defining label and clothing parsing categories that may not be available in real-world scenarios. Alternatively, the authors of [14] proposed a Grid Search Network (GSN) to generate visual embeddings for fashion retrieval. They also used a reinforcement learning based strategy to improve performance and learn a special transformation function over the GSN feature embedding. They generated a target grid by randomly selecting positive and negative patterns with respect to the query image, and then optimized a distance-based grid search loss to enable simultaneous comparison of multiple feature embeddings. The performance of GSN depends heavily on the effective selection of positive and negative samples. In [11], the Siamese-based networks called Graph Reasoning Network (GRNet) were recommended for similarity learning between a query and a gallery clothing by using both global and local representations in different local clothing regions and scales based on a graph convolutional neural network. Another study, [10], employed two neural networks with different parameters to detect the differences between consumer and shop clothing images. However, using two different sets of parameters leads to an increase in the number of parameters, which is not conducive to neural network optimization [15]. In contrast, we perform the cross-domain consumer-to-shop clothes retrieval via the Siamese networks, which have the same weights for both subnetworks. To overcome the limitations of the data problem and avoid the complexity of the network structure to extract stronger features, a novel Discriminative Margin Loss (DML) suitable for apparel search is proposed. The network is optimized with DML to learn discriminative features and achieve more accurate matching.

### 2.2. Loss Functions

Deep Embedding Learning is undoubtedly considered as one of the interesting and significant aspects of the research fields in deep convolutional neural networks, and recently researchers have shown an increasing interest in this area. Loss functions play an important role in deep embedding learning. Deep embedding learning methods increase discriminative power by improving loss functions. Contrastive loss [19,20] and discriminative loss [21] optimize the Euclidean distance of input pairwise samples within a margin for interclass in a feature space. Triplet Loss [22] constructs input triplet samples to separate the positive pair from the negative pair by a Euclidean distance margin for better interclass feature embedding. Therefore, both contrastive loss and Triplet Loss enforce a Euclidean margin for learned features. These methods depend on the number of positive and negative input pairs or triplet images. Therefore, the performance of these loss functions is sensitive to the introduction of pair or triplet mining procedures, which are time consuming [23].

To exploit the supervision property and improve the discriminative power of the deep-learned features, most recent approaches combine Euclidean margin-based losses with softmax losses. For example, Ref. [24] proposed a center loss to learn centers for deep features such that each class minimizes the within-class variations and the given centers are combined with softmax loss. The deep features learned with softmax loss have an intrinsic angular distribution, and Euclidean margin-based losses are not compatible with softmax losses. To address this issue, the researchers decided to optimize the softmax loss for within-class variation. One study, ref. [25], proposed a large margin softmax (i.e., L-Softmax) by adding angle constraints to each identity to improve feature discrimination. Moreover, ref. [23] improved L-Softmax by normalizing the weights and proposed Angular Softmax (A-Softmax). Due to the difficulty of optimizing angle constraints, Refs [18,26,27] moved the angle range to a cosine space and proposed CosFace and ArcFace, respectively. CosFace and ArcFace assign the same decision space to the negative class and the positive class, respectively. In consumer-to-shop fashion retrieval, negative pairs with small visual differences could be considered as positive pairs and affect the retrieval performance. Thus, assigning an equal decision margin to positive and negative classes causes the system to perform poorly on negative pairs with small visual differences. These pairs require a larger decision margin to distinguish them as well as possible from the positive pairs. In contrast to existing loss functions, we propose a novel cross-domain loss that introduces two different margins into the negative and positive interclasses to extract discriminative deep features.

## 3. The Proposed Approach

In this section, we describe the proposed method in detail. First, we discuss the drawbacks of the existing loss functions for the cross-domain problem and explain our motivation for introducing a novel loss function (Section 3.1). The proposed Cross-Domain Discriminative Loss (DML) is presented in Section 3.2. Finally, to better understand the difference between DML and the other loss functions, a visual comparison is made in Section 3.3.

### 3.1. Motivation

Margin-based softmax losses have achieved significant improvements by setting *m* for all the classes to squeeze the intraclass variations. They assumed that the feature distributions of all the classes are identical, so that setting the same margin is enough to constrain all the classes. Since they consider the same margin for the positive and negative pairs, they are not suitable for cross-domain fashion search. For the negative class with large visual differences, the extracted features are placed in the feature distribution of negative samples, but for those negative classes with small visual differences, extracted features may be placed in the feature distribution of the positive class.

**Figure 2:**
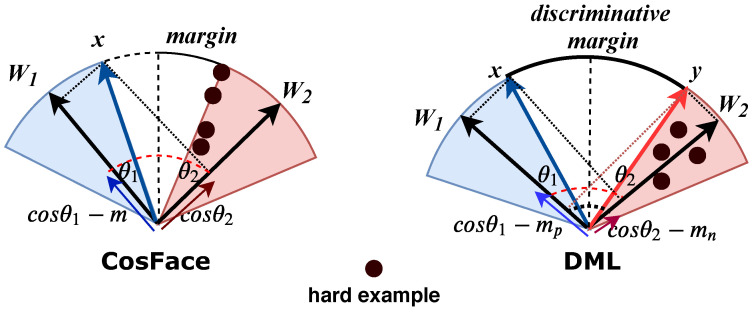
Geometrical interpretation of DML is illustrated from feature perspective. Blue and red areas represent the feature space of the positive and negative classes, respectively. The extracted feature vectors of the positive or negative image pairs are merged into a single vector at the feature level. CosFace [18] sets the same margin *m* for positive and negative classes, so the discrimination process cannot be strong enough. Compared to positive class margin $m_{p}$, DML learns a larger margin $m_{n}$ for the negative class, consequently expands the variations between classes and condenses the variations within classes, implicitly optimizing the discrimination space. Negative pairs with small visual differences move closer to negative pairs with large visual differences, pushing hard examples into the feature space of the negative class.

If a uniform margin *m* is set for the positive and negative classes, the feature distributions of the negative class may not be as compact as those of the positive class. The goal is to achieve a small intraclass for the negative pairs in addition to increasing the variation between classes. If the same margin is considered for the positive and negative classes, the negative pairs that are very similar can be considered as positive, which reduces the functionality of the system in the discrimination process. We further visualize the phenomenon through the process of distinguishing the positive pairs from the negative pairs as shown in Figure 2. Suppose that the normalized feature vectors x and y are given for the positive and negative pairs, respectively. In our work, feature fusion of a pair of images is achieved by adding the deep feature vectors of the two images. The blue region represents the region of positive pairs, while the red region represents the region of negative pairs. In addition, the white region represents the variation between classes. Let $\theta_{1}$ ($\theta_{2}$) denote the angle between the learned feature vector (representing a given pair of images) and the normalized weight vector $w_{1}$ ($w_{2}$). $w_{1}$ and $w_{2}$ are the centers of the positive and negative classes, denoted by $C_{1}$ and $C_{2}$, respectively. The CosFace forces $\cos\left(\theta_{1}\right)-m=\cos\left(\theta_{2}\right)$ for $C_{1}$, and similarly for $C_{2}$, so that features from the positive and negative classes are equally compacted. In a desirable discrimination process, we not only want to maximize the variation between the classes, but also want to minimize the intraclass variation of the negative class. To address this problem, we introduce a novel discriminative margin loss for cross-domain fashion retrieval. By learning a larger margin $m_{n}$ to the negative class compared to the positive class margin $m_{p}$, we simultaneously increase the interclass variation and decrease the intraclass variation of the negative class, ensuring that no very similar negative pairs (hard examples) occur in the positive decision margin.

### 3.2. Cross-Domain Discriminative Margin Loss (Dml)

In Siamese networks, two input images are simultaneously fed into two subnetworks (with the same architecture and weights) and the similarity of the two images is evaluated by the contrastive loss. The contrastive loss is used to train the network to distinguish between similar and dissimilar pairs of examples.

The Siamese network problem is sensitive to calibration because it requires a context for the notion of similarity or dissimilarity [28]. To obtain a robust discriminative model, positive and negative pairs must be introduced with a high number, which is a time-consuming process. Moreover, negative pairs in the loss function cooperate only when their distance is at the decision boundary. On the other hand, the choice of an appropriate value for the decision margin depends on the number and influence of the positive and negative pairs.

To overcome these problems, we merge the embedded features of the two subnetworks and use the softmax function instead of the Euclidean distance, and propose a novel Cross-Domain Discriminative Margin Loss (DML) for cross-domain fashion retrieval. Softmax separates features from different classes by maximizing the posterior probability of the corresponding class. Given the feature vector $x_{i}$ and the corresponding label $y_{i}$, the softmax loss is defined as follows:

(1) $$L_{s}=\frac{1}{N}\sum_{i=1}^{N}-\log p_{i}=\frac{1}{N}\sum_{i=1}^{N}-\log\frac{e^{w_{y_{i}}^{T}x_{i}+b_{y_{i}}}}{\sum_{j=1}^{C}e^{w_{j}^{T}x_{i}+b_{j}}},$$

where $p_{i}$ denotes the posterior probability that the feature vector $x_{i}$ (a single vector formed by fusing the extracted feature vectors of the positive or negative image pairs at the feature level) is correctly classified into the corresponding class $y_{i}$, $w_{j}$ denotes the *j*-th column of the weight matrix W, *b* is the bias term, *N* is the number of training samples, and *C* is the number of classes. Normalizing $x_{i}$ and $w_{j}$ using $L_{2}$ normalization, rescaling $x_{i}$ to *s*, and fixing the bias term b=0, the feature distance is projected onto the feature angle measure for simplicity as follows:

(2) $$L_{s}=\frac{1}{N}\sum_{i=1}^{N}-\log p_{i}=\frac{1}{N}\sum_{i=1}^{N}-\log\frac{e^{w_{y_{i}}^{T}x_{i}+b_{y_{i}}}}{\sum_{j=1}^{C}e^{w_{j}^{T}x_{i}+b_{j}}},$$

where $p_{i}$ indicates the posterior probability of feature vector $x_{i}$ (one single vector which is formed by the fusion of the extracted feature vectors of the positive or negative image pairs at the feature level) being correctly classified into related class $y_{i}$, $w_{j}$ denotes the *j*-th column of the weight matrix W, *b* is the bias term, *N* is the number of training samples and *C* is the number of classes. By normalizing $x_{i}$ and $w_{j}$ using $L_{2}$ normalization, rescaling $x_{i}$ to *s* and fixing the bias b=0 for simplicity [18], the feature distance is projected to feature angular as follows:

(3) $$w_{j}^{T}x_{i}=∥w_{j}∥∥x_{i}∥\cos\theta_{ji}=s\;\cos\theta_{ji},$$

where $\theta_{ji}$ is the angle between $w_{j}$ and $x_{i}$. Thus, both the norm and the angle of the vectors contribute to the posterior probability. Based on this formulation, some methods have been proposed to optimize and extend the interclass margin [18,26]. Since optimization in cosine space is much easier compared to angle space, we further focus on the analysis of cosine margin. By importing the margin *m* into the cosine space of Softmax, the Large Margin Cosine Loss (LMCL) [18] attempts to further distinguish it as follows:

(4) $$L_{lmc}=\frac{1}{N}\sum_{i=1}^{N}-\log\frac{e^{s\left(\cos\left(\theta_{y_{i},i}\right)-m\right)}}{e^{s\left(\cos\left(\theta_{y_{i},i}\right)-m\right)}+\sum_{j\neq y_{i}}^{C}e^{s\cos\left(\theta_{j,i}\right)}},$$

subject to

(5) $$\cos\left(\theta_{j,i}\right)=w_{j}^{T}x_{i},$$

where *N* is the number of training samples, $x_{i}$ is the *i*-th feature vector corresponding to the ground truth class of $y_{i}$, $w_{j}$ is the weight vector of the *j*-th class, and $\theta_{j,i}$ is the angle between $w_{j}$ and $x_{i}$.

Since cross-domain fashion retrieval is a discriminative binary problem, we have only two classes (similar and dissimilar classes). Therefore, $\theta_{1}$ and $\theta_{2}$ denote the angles between the embedding feature vectors and the weight vectors of class $C_{1}$ and $C_{2}$, respectively. In the LMCL method, the value of the margin *m* is considered as a constant value for positive and negative classes, resulting in pairs with small visual differences (hard examples) being identified as positive pairs. This problem is particularly prevalent in cross-domain fashion retrieval, where there is a high degree of similarity in design and appearance between different types of clothing. Our goal is to expand the variation between classes to distinguish negative pairs from positive pairs and condense the negative feature space to gather negative pairs with small and large visual differences. This prevents hard examples from entering the feature space of positive pairs and increases the discriminative power. To this end, we do not assign the same margin *m* to the negative class and the positive class, but assign a larger *m* to the negative class to reduce the intraclass variation of the negative class. For clarity, we represent the angles below with only one subscript corresponding to the class. In other words, $\theta_{j,i}$ is denoted by $\theta_{j}$. For the positive class, and similarly for the negative class, the cross-domain loss is formulated as follows:

(6) $$L_{Cross-Domain}=\frac{1}{N}\sum_{i=1}^{N}-\log\frac{e^{s\left(\cos\left(\theta_{y_{i}}\right)-m_{y_{i}}\right)}}{e^{s\left(\cos\left(\theta_{y_{i}}\right)-m_{y_{i}}\right)}+e^{s\left(\cos\left(\theta_{j}\right)\right)}},$$

where *N* is the number of training samples, $m_{y_{i}}$ is the margin assigned to the ground truth class $y_{i}\;in\left\{p,n\right\}$ of the *i*-th pair (where for the positive class is $m_{p}$ and for the negative class is $m_{n}$), and $j\neq y_{i}$. $m_{n}$ should be larger than $m_{p}$. Setting $m_{n}>m_{p}$ aims to compact the negative decision boundary and expand the interclass and reduce the negative intraclass, which also ensures the absence of the hard examples in the positive feature space.

To ensure the discriminative power of cross-domain loss and provide a decisive solution, we introduce the discriminative part as follows:

(7) $$L_{discriminative}=-\left(\lambda_{1}\times m_{p}+\lambda_{2}\times m_{n}\right)/2,$$

where $\lambda_{1}$ and $\lambda_{2}$ ($\lambda_{1}<\lambda_{2}$) are balancing factors to control the size of the positive and negative margins. By combining (5) and (6), the cross-domain discriminative margin loss (DML) is proposed as follows:

(8) $$\begin{array}{c} L_{DML}=L_{Cross-Domain}+L_{discriminative}=\\ \frac{1}{N}\sum_{i=1}^{N}-\log\frac{e^{s\left(\cos\left(\theta_{y_{i}}\right)-m_{y_{i}}\right)}}{e^{s\left(\cos\left(\theta_{y_{i}}\right)-m_{y_{i}}\right)}+e^{s\left(\cos\left(\theta_{j}\right)\right)}}-\left(\lambda_{1}\times m_{p}+\lambda_{2}\times m_{n}\right)/2, \end{array}$$

where $m_{p}$, $m_{n}$ are the margins for positive and negative classes, $\theta_{y_{i}}$ is the angle between $x_{i}$ (the fused feature vector of the positive or negative pair) and the vector $w_{y_{i}}$. The hyperparameters $\lambda_{1}$ and $\lambda_{2}$ control the discriminative power of DML.

### 3.3. Comparison to Other Loss Functions

**Figure 3:**
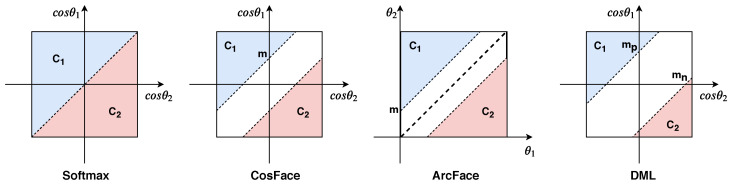
The decision margin of different loss functions for discriminative analysis is visualized. $C_{1}$ is a positive class and $C_{2}$ is a negative class. Blue, red, and white areas represent positive decision margin, negative decision margin, and decision limit, respectively. As can be seen, unlike other losses that consider constant margins *m* for the positive and negative decision margins, DML learns margins $m_{p}$, $m_{n}$ for the positive and negative decision margins, where $m_{n}>m_{p}$.

To better understand the advantages of DML over existing losses, the decision boundary for the discrimination problem is shown in Figure 3. Softmax considers margin=0 between the positive class $C_{1}$ and the negative class $C_{2}$. CosFace and ArcFace specify a constant value for the margin between positive and negative classes. We argue that these strategies are not suitable for clothing analysis because the distribution of the negative class is not uniform, i.e., negative pairs can have both small and large visual differences.

To overcome this challenge in cross-domain fashion search, the proposed loss assigns a learnable margin to each class, while a larger margin is enforced for the negative class. The larger margin $m_{n}$ compacts scattered negative pairs with small and large visual differences and shifts the decision boundary of the negative class $C_{2}$ away from the positive class $C_{1}$.

Since the number of negative pairs is higher than the number of positive pairs (due to the limited amount of data), consumer-to-shop fashion retrieval could be considered as a class imbalanced problem, where the training can be dominated by the most frequent class (negative pairs). FCdDN [29] proposed a loss function to reassign the probability value of the dominant class to a smaller value to overcome this problem. Specifically, FCdDN maps the probability values of the dominant class (negative pairs) to a smaller value and the probability values of the poor class (positive class) to a larger value. By focusing attention on the dominant class and giving it more weight, FCdDN attempts to solve the imbalance problem. Compared to FCdDN, DML not only tries to solve the imbalance problem by assigning a larger margin to the negative class, but also tries to prevent the positive margin from becoming equal to the negative margin due to the discriminative part, which leads to distinguish between hard examples and positive pairs.

## 4. Experiments

### 4.1. Datasets

We evaluated our proposed method with the dataset DARN and with two benchmarks of the DeepFashion dataset: (1) InShop Clothes Retrieval and (2) Consumer-to-Shop Clothes Retrieval.

The DARN dataset was collected specifically for street-to-shop retrieval and contained approximately 327,000 in-shop images and 91,000 user images. Since the collectors of the DARN dataset did not provide a standard protocol and the files provided by the authors contain broken links, we use the cleaned version provided by [6,10] and follow their evaluation protocol for a fair comparison. First, they removed corrupted images to obtain a subset of 62,812 street images and 238,499 shop images of 13,598 distinct products distributed over 20 fashion categories where each street image has a matched shop image. Then, they partitioned the dataset into three subsets for training, validation, and test, with no overlap of products (see Table 1).

The DeepFashion dataset [16] is one of the largest datasets for clothing image analysis and contains more than 800k images. Each image in this dataset is annotated with labels of categories, attributes, bounding boxes, and landmarks. The presence of occlusions, deformations, lighting variations, and large variations in pose and scale have made this dataset challenging. The Consumer-to-Shop Clothes Retrieval benchmark contains 239,557 consumer-to-shop images with 33,881 clothing items. The InShop Clothes Retrieval benchmark contains 52,712 images with 7982 garments. Their partitions are shown in Table 1. Note that in the InShop benchmark, the gallery set images are used as training shop photos and the query set images are used as the test shop photos. To ensure a fair comparison, the split between training and testing is given. Consistent with the state of the art, we used this split in all of our experiments. In addition, each image was cropped using the bounding boxes provided.

### 4.2. Implementation Details

**Figure 4:**
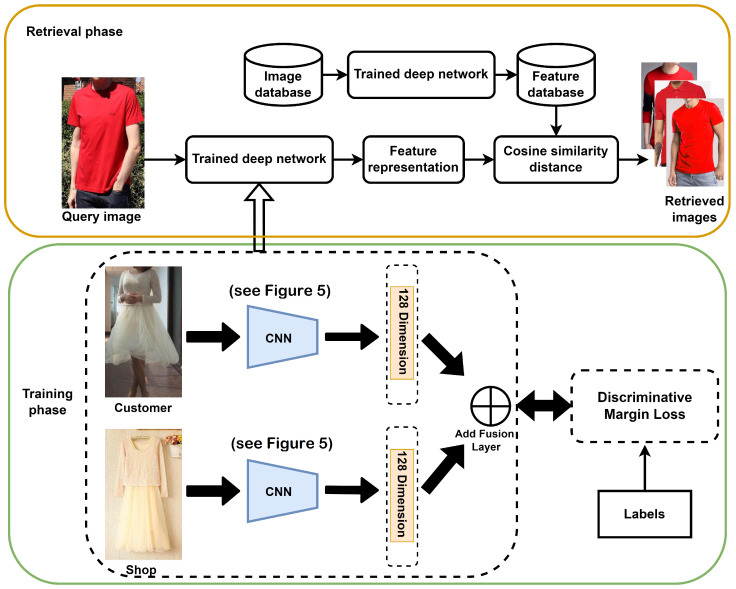
The overview of our proposed cross-domain consumer-to-shop clothes retrieval system. The Siamese network consists of two subnetworks with the same architecture and weights. The extracted features of the two subnetworks are normalized by $L_{2}$ normalization. The two 128-dimensional embedding instances for customer and shop images are merged by Add fusion layer. The DML loss drives the training of the network to learn features where the discriminative decision boundary increases and the negative margin becomes more compact. Then, the trained deep network is used to extract features from the image database and create a feature database. In the retrieval phase, features of the query image are extracted by the trained deep network and compared with the features of the feature database by the cosine similarity distance. Finally, top ranked results are displayed to the customer.

**Figure 5:**
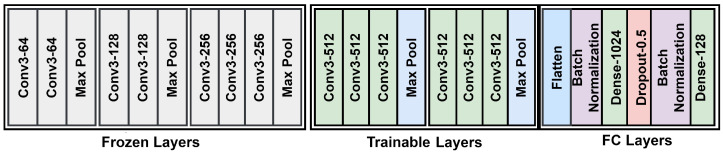
The proposed CNN architecture is based on the VGG16 network. The weights of this net are pre-trained on ImageNet dataset. The fully connected layers are changed in our architecture. The weights of the first three groups are frozen and the weights of the last two groups are trained using the datasets.

The propsoed Siamese network is shown in Figure 4. A Siamese network contains two identical CNNs, one for shop images and one for customer images. We considered a VGG-16 architecture for each subnetwork trained on the ImageNet dataset. The architecture of this model is shown in Figure 5. A 128-dimensional feature vector was extracted from each network and normalized with $l_{2}$ norm, then these two features extracted from the two subnetworks were combined by Add Fusion Layer. Stochastic gradient descent (SGD) was used to optimize the network. We used the initial learning rate $1\times 10^{-4}$ and the weight decay as $5\times 10^{-4}$. We followed [18] to set the feature scale *s* to 64 and the momentum to 0.9. We chose the initial angular margins $m_{n}$ and $m_{p}$ to be 0.4 and 0.35, respectively. We empirically found that when $\left(\lambda_{1},\lambda_{2}\right)=\left(70,75\right)$, the system reached its highest performance (see Section 4.5). The model and loss layer were implemented in Python 3.6 using the deep learning library Keras 2.2.4 and trained with a batch size of 128 on an NVIDIA GeForce RTX 2080 Ti GPU. In the testing phase, the model was used to extract the feature vector from the customer and shop images, and its similarity is calculated by the cosine distance. The retrieval performance of the proposed method is evaluated by the top-k accuracy as in [16], i.e., the ratio of correct matches (in the set of queries) within the top-k results.

### 4.3. Experimental Results

In this section, we compare our proposed method with the state of the art in three public benchmarks for fashion product retrieval. Note that the contributions of the baseline solutions and our method are orthogonal. Compared to baselines, we focus on discriminative analysis by proposing a new loss function DML and evaluating the contribution of DML with pretrained VGG16. We can use attention module based architectures to further improve our model. DeepFashion has introduced a standard protocol with training, validation, and testing sets. We followed the standard protocol and evaluated our approach on two benchmarks from DeepFashion: InShop Clothes Retrieval and Consumer-to- Shop Clothes Retrieval. Table 2 compares the proposed DML with state-of-the-art methods, including FashionNet [16], Siamese-Triplet [8], VAM+ImgDrop [8], DREML [30], KPM [31], AHBN [32], and GRNet [11] on Consumer-to- Shop Clothes Retrieval. GRNet performed best among the state-of-the-art methods for Top-1 and Top-20. Note that GRNet’s contribution is to use both global and local representations at multiple scales, which is orthogonal to our method. Clearly, we can use GRNet to further improve our model. As for the comparison of the proposed method with the other approaches, DML improves the retrieval performances for Top-1, Top-20, and Top-50 by 2.3, 8.3, and 10.7%, respectively.

**Figure 6:**
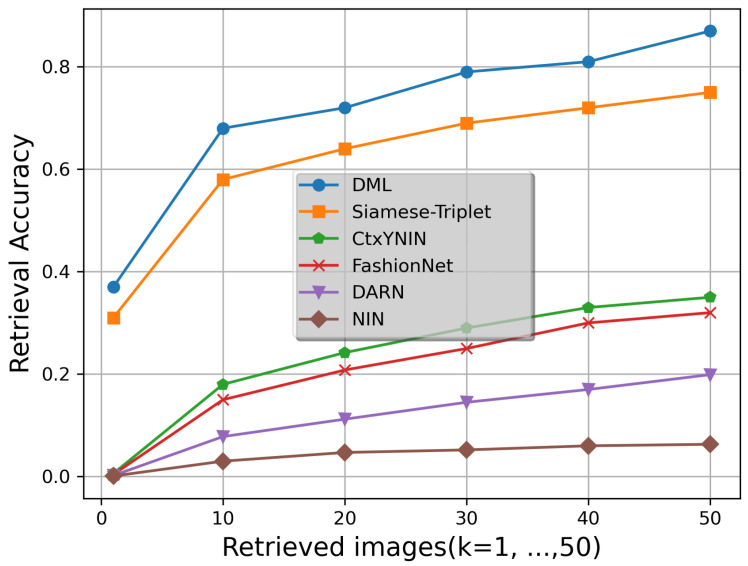
Top-k accuracy rates for different methods under comparison on DARN Consumer-to-shop retrieval dataset. The last four methods are reported by [6] and Siamese-Triplet is reported by [10].

To evaluate and demonstrate the effectiveness of the proposed method for images from the same domains, we evaluated InShop Clothes Retrieval. As can be seen in Table 3, our approach achieves the best top-1 accuracy of 0.712. For top-20 and top-50, our approach achieves an accuracy slightly lower than the performance of VAM. It is worth noting that VAM uses an attention subnetwork that requires a clothing segmentation dataset for training, while DML is trained using only image pairs from queries and galleries, which is more practical. We also evaluate our method using the DARN dataset. The results are shown in Figure 6. Due to a different task and dataset, the pretrained NIN performs the worst. DARN and FashionNet models perform better than NIN because they consider tag information during training. Unlike DARN and FashionNet, CtxYNIN uses tags such as product category and semantic attributes not only in training but also in the query phase, which helps to draw attention to the shop images when the background is noisy. As shown in Figure 6, Siamese-Triplet has the best performance among the previous methods, indicating that the Siamese architecture significantly improves the retrieval performance compared to a single model. Since the Siamese-triplet method is coupled with a triplet loss function to optimize the network, it requires a large number of input pairs for learning. As mentioned earlier, collecting and annotating sufficient data is a major challenge in fashion analysis. DML outperformed state-of-the-art methods and improved fashion retrieval performance by giving the negative class a relatively larger margin than the positive class. It can be seen that DML and Siamese-Triplet retrieval performances are close to each other from Top-1 to Top-10. After Top-10, the retrieval performance of Siamese-Triplet increases with a relatively constant slope, indicating the limited ability of Triplet Loss to distinguish fashion positive pairs from negative ones. In contrast, DML retrieval performance increases nonlinearly and shows considerable improvements in Top-30 and Top-50.

Due to the larger scale, variety and quantity of image clothing of the DeepFashion dataset compared to the DARN dataset, it can be seen that the retrieval results of the different methods on the DeepFashion dataset are better than those on the DARN dataset (see Table 2 and Figure 6).

### 4.4. Comparison with Other Loss Functions

**Figure 7:**
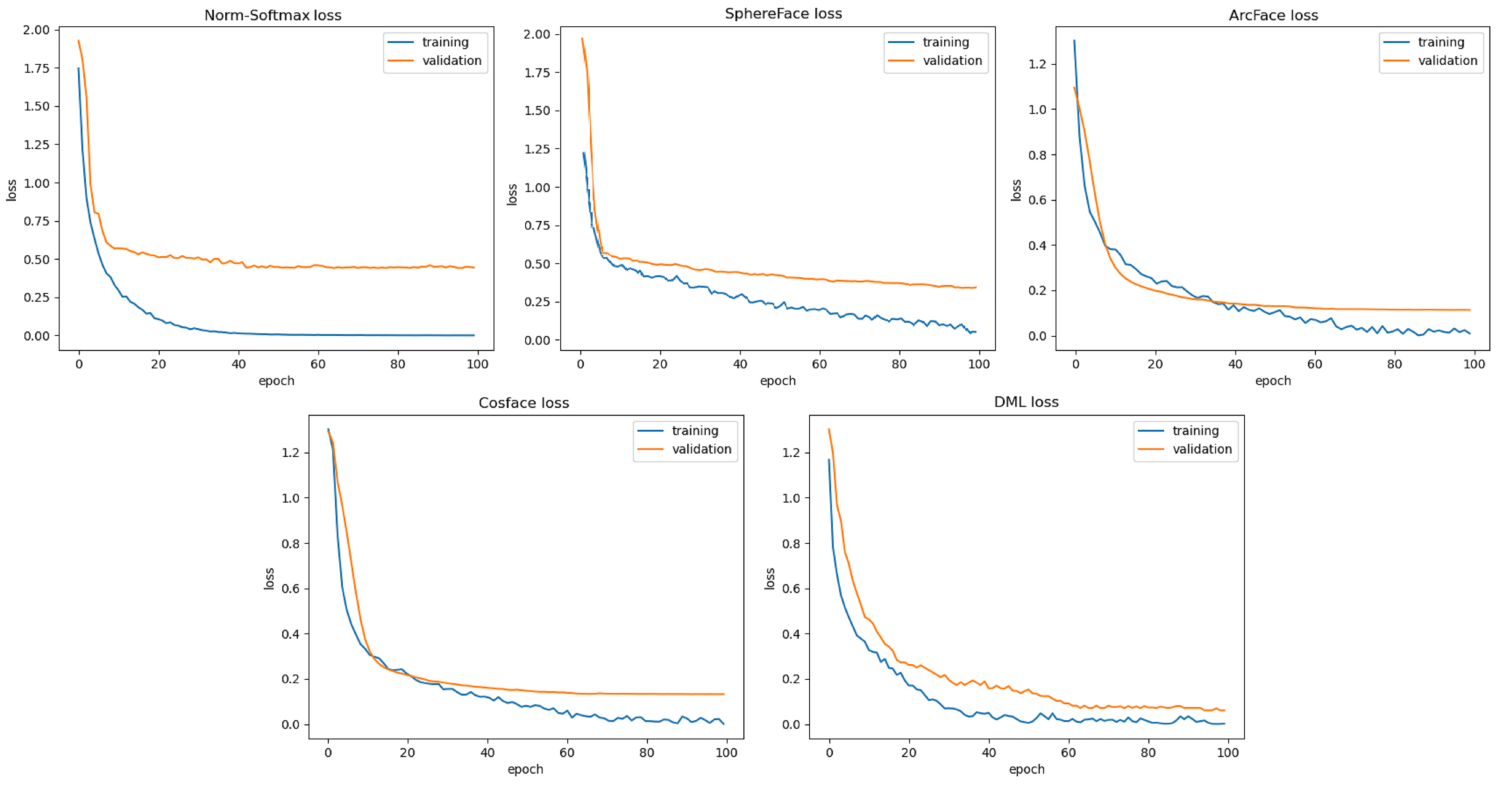
Training and validation losses for various margin-based Softmax loss functions using VGG16 network discussed in Section 4.2 for the consumer-to-shop clothes retrieval benchmark of the DeepFashion dataset. These results indicate that using DML for training leads to lower training loss than all other margin-based Softmax losses for consumer-to-shop clothes retrieval.

To show the main contribution of our approach in cross-domain problems, we compare the performance of the proposed DML with state-of-the-art margin-based Softmax losses such as Norm-Softmax, SphereFace, ArcFace, and CosFace. According to the implementation details in Section 4.2, we train our Siamese networks on the DeepFashion and DARN datasets with the same CNN architecture and different loss functions. Since the backbone CNN of the two subnetworks is fixed, the difference in performance is due to the losses used. According to the literature, the best performances of the SphereFace, ArcFAce, and CosFace methods are obtained with margin values of 1.35, 0.50, and 0.35, respectively. Table 4 shows the retrieval performance (top-20) of different loss functions on DeepFashion and DARN. Norm-Softmax was obtained by normalizing features and weights which consequently has less discriminative power due to the lack of margin. SphereFace improves angular discrimination by using a multiplicative angular margin, but it requires a series of approximations to be computed, resulting in an unstable training of the network. ArcFace and CosFace directly add an angular margin and a cosine margin penalties to the target logit, respectively, resulting in better performance compared to SphereFace, but they set the same decision margin for the negative and positive classes, causing the system to perform poorly on negative pairs with small visual differences. As shown, DML achieves competitive results compared to the other margin-based Softmax losses on both datasets. In particular, our loss function significantly outperforms margin loss functions such as CosFace and ArcAFce, which attempt to extend the decision boundary and distinguish positive and negative pairs. Due to the larger margin set for the negative class compared to the positive class, the decision boundary between positive and negative decision margins expands more and the negative pairs with small and large visual differences move as close as possible. The training and validation losses for various margin-based softmax loss functions using the VGG16 network discussed in Section 4.2 are shown in Figure 7 for the consumer-to-shop clothes retrieval benchmark of DeepFashion dataset. The results in Figure 7 show that DML significantly outperforms the other loss functions in reducing training and validation losses.

### 4.5. Effects of $\lambda_{1}$ and $\lambda_{2}$ on Discriminative Margin Loss

**Figure 8:**
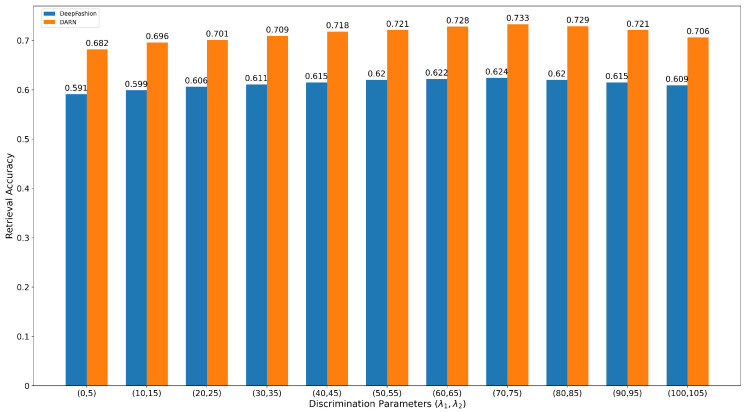
The retrieval performance of Discriminative Loss Margin with different discrimination parameters $\lambda_{1}$ and $\lambda_{2}$ in Consumer-to-Shop Clothes Retrieval (top-20).

Discriminative Margin Loss consists of two parts, the cross-domain loss, and the discriminative margin average loss. The discriminative part of DML plays an important role in preventing the positive margin $m_{p}$ from becoming equal to the negative margin $m_{n}$ during the training process. In this part, we conduct an experiment to investigate the effects of the different combinations of $\lambda_{1}$ and $\lambda_{2}$. By varying the value of $\lambda_{1}$ from 0 to 100 and $\lambda_{2}$ from 5 to 105, we obtain different combinations of $\lambda_{1}$ and $\lambda_{2}$. Then, we train our model on DeepFashion and DARN training subsets and validate it on the test subsets. Since our ultimate goal is to make $m_{n}$ larger than $m_{p}$, we set the value of $\lambda_{2}$ above $\lambda_{1}$. As shown in Figure 8, the retrieval performances on Consumer-to-Shop Clothes Retrieval benchmark of DeepFshion and DARN improves with the increase of $\lambda_{1}$ and $\lambda_{2}$ from 0 to 70 and from 5 to 75, respectively. When $\left(\lambda_{1},\lambda_{2}\right)=\left(70,75\right)$, the system appears to reach its highest performance and enters saturation, after which system performance begins to decline. It is evident that DML can learn the decision boundaries of the positive and negative classes to deal with the small visual differences of the negative pairs.

## 5. Discussion

In this work, we addressed the important role of discriminative analysis in cross-domain consumer-to-shop clothes retrieval. Previous methods proposed complex architectures that are highly computationally intensive, resulting in uncertain real-time performance. Unlike previous methods that attempted to improve retrieval performance by optimizing CNN structures to extract local and global features, we aimed to improve loss-function performance. To this end, we proposed a novel loss function called Discriminative Margin Loss (DML) to enforce a small intraclass distance and increase the distance between input pairs labeled as dissimilar. Evaluation of the retrieval performance of DML in three public fashion product retrieval benchmarks showed that DML performed best. Nevertheless, better performance can also be achieved by using a previous complex feature-extraction architecture. Compared to previous methods, DML has two advantages. First, it provides high retrieval performance when trained only on image pairs of query and gallery, which is more practical. Second, it is insensitive to the constraints of the data problem. The proposed loss function has several strengths that are not found in other margin-based softmax loss functions. These are as follows. First, DML does not assume the same margin for positive and negative pairs, resulting in more negative pairs being compressed than positive pairs. Second, if $x_{i}$ deviates too much from the center $W_{y_{i}}$, assigning different margins for positive and negative regions results in part of the overlap region not being recognized as positive class and negative class. The proposed loss function is generic in the sense that it can be easily extended to the verification and binary classification problems. Similar to the existing margin-based loss functions, the major limitation of the DML is that its performance depends on the process of tuning the hyperparameters. We should explore different sets of numbers to find the best margins and $\lambda_{1}$ and $\lambda_{2}$ depending on the problem. This means that the best parameters for the cross-domain consumer-to-shop retrieval problem would not suitable for another problem such as face verification.

## 6. Conclusions

In this work, a loss function called DML is proposed to improve the performance of CNNs in consumer-to-shop clothes retrieval. Unlike existing margin-based softmax losses, DML learns two different margins for negative and positive classes to increase compactness within classes and separability between classes. The margin for negative classes is larger than the margin for positive classes. Accordingly, DML attempts to increase cross-class separability and focuses on negative intraclass compactness. For this reason, negative pairs with small visual differences are not considered as positive pairs, resulting in improved retrieval performance. Extensive experimental results on three public fashion datasets show significant advantages over state-of-the-art methods and all compared margin-based softmax functions. According to the results, DML was the most successful to retrieve clothes and achieved Top-50 retrieval performances of 0.759, 0.921, and 0.87 on the Consumer-to-Shop Clothes Retrieval benchmark, the InShop Clothes Retrieval benchmark, and DARN dataset, respectively. Future research directions include: (1) improving the performance of the CNN used or replacing it with other Deep Learning architectures such as GRNet to leverage both global and local representations at multiple scales; (2) generalizing DML to the multiple-class scenario to strengthen the discrimination of learned features by promoting a specific additional margin for each class in cosine space.

## Figures and Tables

**Table 1:** The data splitting of DARN, Consumer-to-Shop, and InShop datasets.

	Dataset		
	DARN	DeepFashion: Consumer-to-Shop	DeepFashion: InShop
Distinct Training Products	10,979	15,898	3997
Training Street Photos	50,528	98,768	-
Training Shop Photos	32,194	98,768	25,882
Number of positive pairs	50,528	98,768	13,528
Number of negative pairs	252,640	493,840	67,640
Distinct Validation Products	9635	8076	-
Validation Street Photos	6318	48,917	-
Validation Shop Photos	23,828	48,917	-
Distinct Test Products	9636	8077	3985
Test Street Photos	5966	47,734	-
Test Shop Photos	23,773	47,734	26,830

**Table 2:** Comparison of top-k accuracy rates on Consumer-to-Shop Clothes Retrieval benchmark of the DeepFashion dataset. Bold shows the the best rate.

	Accuracy		
Method	Top 1	Top 20	Top 50
FashionNet [16]	0.073	0.188	0.228
Triplet [8]	0.109	0.378	0.499
VAM+ImgDrop [8]	0.137	0.439	0.569
DREML [30]	0.186	0.510	0.591
KPM [31]	0.213	0.541	0.652
AHBN [32]	-	0.603	-
GRNet [11]	**0.257**	**0.644**	0.750
DML	0.236	0.624	**0.759**

**Table 3:** Comparison of top-k accuracy rates on the InShop Clothes Retrieval benchmark of the DeepFashion dataset. Bold shows the the best rate.

	Accuracy		
Method	Top 1	Top 20	Top 50
FashionNet [16]	0.529	0.764	0.796
VAM [8]	0.669	**0.892**	**0.945**
DARN [6]	0.382	0.675	0.717
Diversity Fashion [33]	-	0.784	-
Studio2Shop [34]	-	0.818	-
GoogleNet [8]	0.554	0.823	0.877
DML	**0.712**	0.875	0.921

**Table 4:** Comparison of the proposed DML with state-of-the-art margin-based loss functions in Consumer-to-Shop Clothes Retrieval (top-20). All methods in this table used the same training data and the same Siamese network architecture. Bold shows the the best rate.

		Accuracy	
	Dataset	DeepFashion	DARN
Loss			
Norm-Softmax		0.32	0.46
SphereFace (m = 1.35)		0.55	0.59
ArcFace (m = 0.50)		0.57	0.61
CosFace (m = 0.35)		0.58	0.64
DML		**0.62**	**0.73**

## Data Availability

Not applicable.
